# Immune contexture analysis in immuno‐oncology: applications and challenges of multiplex fluorescent immunohistochemistry

**DOI:** 10.1002/cti2.1183

**Published:** 2020-10-07

**Authors:** Reshma Shakya, Tam Hong Nguyen, Nigel Waterhouse, Rajiv Khanna

**Affiliations:** ^1^ QIMR Berghofer Centre for Immunotherapy and Vaccine Development, Tumour Immunology Laboratory QIMR Berghofer Medical Research Institute Brisbane QLD Australia; ^2^ Flow Cytometry and Imaging Facility QIMR Berghofer Medical Research Institute Brisbane QLD Australia

**Keywords:** FCS express image cytometry, immune profiling, multiplexed fluorescent immunohistochemistry, quantitative digital pathology, tumor microenvironment, vectra

## Abstract

The tumor microenvironment is an integral player in cancer initiation, tumor progression, response and resistance to anti‐cancer therapy. Understanding the complex interactions of tumor immune architecture (referred to as ‘immune contexture’) has therefore become increasingly desirable to guide our approach to patient selection, clinical trial design, combination therapies, and patient management. Quantitative image analysis based on multiplexed fluorescence immunohistochemistry and deep learning technologies are rapidly developing to enable researchers to interrogate complex information from the tumor microenvironment and find predictive insights into treatment response. Herein, we discuss current developments in multiplexed fluorescence immunohistochemistry for immune contexture analysis, and their application in immuno‐oncology, and discuss challenges to effectively use this technology in clinical settings. We also present a multiplexed image analysis workflow to analyse fluorescence multiplexed stained tumor sections using the Vectra Automated Digital Pathology System together with FCS express flow cytometry software. The benefit of this strategy is that the spectral unmixing accurately generates and analyses complex arrays of multiple biomarkers, which can be helpful for diagnosis, risk stratification, and guiding clinical management of oncology patients.

## Graphical abstract

This review discusses current developments in multiplexed fluorescent immunohistochemistry for immune contexture analysis, and their application in immuno‐oncology, and discusses challenges to effectively use this technology in clinical settings. We also present a multiplexed image analysis workflow to analyse fluorescence multiplexed stained tumor sections using the Vectra Automated Digital Pathology System together with FCS express flow cytometry software.

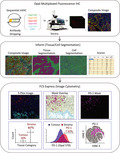

## Introduction

Progression of all solid cancers is directly influenced by complex interactions between immune and non‐immune cells within the tumor microenvironment (TME).^1^, ^2^ Understanding the immune architecture of a tumor is becoming increasingly important for evaluating disease and therapeutic responses, particularly in immunotherapy. Recent investigations have shown a strong link between intra‐tumor infiltration of lymphoid cells with improved clinical outcome and anti‐tumor response.^3^, ^4^ Further investigations of the TME have suggested that the number, type, location and functional profile, also known as ‘immune contexture’ of tumor‐infiltrating lymphocytes in primary tumors, are associated with prognostic benefit.^1^, ^5^, ^6^, ^7^ Therefore, a systems biology approach of integrating immune contexture with clinical outcome may help identify prognostic and predictive biomarkers that will be useful in improving clinical management of patients. One such marker that has received FDA approval for its use as a companion and complementary diagnostic for the therapeutic checkpoint inhibitors pembrolizumab and nivolumab is ‘Programmed cell death ligand‐1 (PD‐L1)’, a transmembrane protein that suppresses the adaptive arm of the immune system.^8^, ^9^ With further developments in the field of cancer therapy, additional biomarkers will be required to predict clinical benefit and improve therapy success.

## Evolving technology for immune contexture analysis

**Figure 1:**
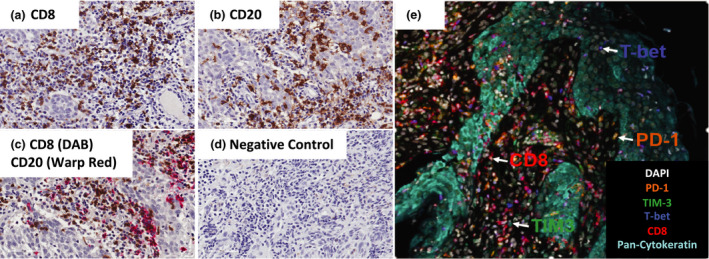
Schematic for chromogenic immunohistochemistry and multiplexed immunofluorescence staining for tumor‐infiltrating lymphocytes. Chromogenic single **(a, b)** staining, double staining **(c)** with negative control **(d)**. Nuclei of all cells are round and stained with CD8 and CD20 expressed on the cell surface (around the nuclei). **(e)** Representative image of nasopharyngeal carcinoma tissue stained with CD8, PD‐1, T‐bet, Tim‐3 and Pan‐cytokeratin using Opal fluorescence multiplexed immunohistochemistry.

Biomarker analysis on a single‐cell basis can be performed by using multi‐parameter detection methods such as genomics/proteomics and flow cytometry on dissociated tissue. However, the spatial information about the location of cells within the tumor core or margin is lost using these technologies.^10^, ^11^, ^12^, ^13^ Until recently, tissue histopathological examination of formalin‐fixed paraffin‐embedded (FFPE) tissue sections using haematoxylin and eosin (H&E) staining has been used to evaluate the morphological changes associated with disease diagnosis and response to therapy.^14^, ^15^ Pathological detection is most commonly performed by evaluating the expression of one or two proteins at a time on serial sections using antigen‐specific antibodies. However, such an approach may be restrictive if the size of the pathogenic area in the resected tissue is small with few detectable tumor cells, and the number of biomarkers to be evaluated is high, because the number of serial sections that can be cut may be insufficient to evaluate all of the required biomarkers. Recent developments in multiplexed immunohistochemistry (mIHC) on FFPE specimen have allowed for simultaneous identification of multiple markers in one tissue section as opposed to a single biomarker on multiple slides. Brightfield multiplexing requires FFPE tissues to be probed with various enzyme/chromogen pairs that results in chromogenic depositions that can be visualised using standard light microscopy (Figure 1a–d).^16^ Although brightfield analyses may be useful to differentiate different cell types, this method is cumbersome when the target proteins are co‐localised within the cells. Additionally, the possibility of primary antibody species cross‐reactivity and chromogenic overlap is also undeniable during multiplexing. Thus, despite the benefit of chromogenic multiplexing in pathological advancement, chromogenic analyses may be practically restricted to measuring less than three proteins on a single slice of tissue.

Multiplex fluorescence IHC (mfIHC) uses fluorescent dyes that offer a greater degree of spectral separation than chromogenic dyes and are commonly used for complex phenotyping of immune cells by flow cytometry. Traditional methodologies for labelling tissue with fluorescent probes involve detecting the protein of interest with an antigen‐specific primary antibody followed by a secondary antibody coupled to an organic fluorescent dye. In some cases, it is also possible to use primary antibodies directly conjugated to fluorescence dyes. These techniques are generally effective for labelling abundant proteins such as CD3 or CD8 but may not be effective for detecting low‐abundance proteins as the signal may not be easily detected above background. Dyes such as nanocrystal quantum dots are reported to be brighter and more stable than organic dyes making it easier to detect low‐abundance proteins.^17^ Regardless of the brightness of the stain, the number of proteins that can be stained and imaged on one tissue is restricted by the position and spread of the different fluorophores along the electromagnetic spectrum. Recent development of new dyes such as Opal dyes with more options for excitation and sharper emission spectra has reduced the potential contribution of spectral overlap in protein detection and image analysis.^18^ Opal dyes use tyramide signal amplification (TSA), a method that enhances the signal of low‐abundance proteins by conjugating several dye molecules to the tyrosine residues of the protein. Further, TSA uses horseradish peroxidase‐conjugated secondary antibodies that are traditionally used for chromogenic IHC making it possible to use TSA to detect proteins that are commonly used in the clinical‐pathological laboratories with little or no changes to tissue handling or processing. The primary and secondary antibodies can also be removed by microwaving the tissue without removing the dyes from the sample, thereby allowing staining of subsequent proteins with different dyes on the same slide, without the risk of nonspecific staining as a result of antibody cross‐reactivity. TSA can therefore be used to accurately label a wider variety of proteins on a single slide than is currently possible with chromogenic or traditional immunofluorescence‐IHC. It is now possible to stain immune cell populations for various markers (e.g. CD3, CD4, CD8, CD20, CD25, CD68, CD69, FOXP3, PD‐1, Tim‐3 and Ki‐67, or any other combination) on a single tissue slice with a multiplex antibody panel to enumerate and evaluate complex phenotypes in the TME (Figure 1e).^18^ To date, several different fluorescence‐based multiplexed IHC techniques have been developed such as tyramide signal amplification, nanocrystal quantum dots, tissue‐based cyclic IF, and MultiOmyx™,^17^, ^18^, ^19^, ^20^, ^21^ that allow labelling of multiple proteins on the same histological sections. These multi‐labelled fluorescent proteins have been commonly used in cell biology to visualise proteins and organelles, using multi‐parameter fluorescence imaging such as confocal microscopy. The sensitivity and reliability of immunofluorescence are compromised by the presence of native (auto)‐fluorescence that is commonly present in formalin‐fixed tissues (FFPE).^22^ Furthermore, fluorescence imaging requires fluorophores to be excited with different wavelengths of light, which requires placement of different filters in sequence during imaging. Thus, one solution to multi‐marker analysis on the same tissue and on the same slide is to employ spectral unmixing, a technique that uses specific characteristics of the dyes to accurately separate out the different staining patterns for each protein.^18^ This technology uses cameras that capture different wavelengths of light in different images allowing the true signal of the dye to be extracted using simple mathematics. Multispectral imaging system (MSI) can be used with sequentially stained single colour chromogenic staining as well as with fluorescence‐labelled samples.^18^, ^23^ The use of MSI with multiplexed fluorescent IHC is particularly more useful for resolving multiple overlapping fluorochromes.

Traditional fluorescence microscopes, even those equipped with multispectral capabilities, have limited scope for movement in the *X* and *Y* planes without moving out of focus, or acquiring large stacks of images in the *z* plane. However, tumors often exhibit a significant degree of cellular and spatial heterogeneity (e.g. stroma, tumor–stroma interface, intra‐tumor). Consequently, there is a growing need for high‐resolution multiplexed analysis across whole tissue sections. As such, the development of microscopes that incorporate MSI and whole slide scanning, or an image stitching feature, can enable researchers to reliably image the whole tumor tissue and quantify multiple proteins, even in the presence of spatial and spectral overlap.^18^ Currently, there are two microscopy companies, Akoya Biosciences (was PerkinElmer) and TissueGnostics, that manufacture and sell fluorescence scanning microscopes with multispectral capabilities (Table 1). The Akoya Biosciences Vectra^®^ automated quantitative pathology imaging system is currently one of the most widely used quantitative digital pathology imaging systems that uses whole slide scanning and multispectral unmixing to resolve overlapping fluorescent signals.^24^, ^25^ Whole slide tissue scanning of multispectral images from multiplexed samples requires longer time to acquire images compared to conventional whole‐slide fluorescence or chromogenic scanners. To overcome this limitation, the Vectra imaging platform utilises lower resolution to scan the whole tissue followed by subsequent MSI of marked fields of view/regions of interest at higher resolution. Nevertheless, such an approach does not fully support high‐resolution whole‐slide analytics.

**Table 1:** Fluorescence multiplexed slide scanning and image analysis platforms

Company	Image acquisition and scanning instrument	Scanner type	Image selection	Supported fluorescence image analysis packages
Akoya Biosciences	Vectra^®^/Vectra^®^ Polaris™	MSI (BF & FL)	ROI, WSI	InForm
TissueGnostics	TissueFAXS PLUS/SPECTRA	BF & FL/MSI (BF & FL)	WSI	StrataQuest
Leica Biosystems	Aperio Versa	BF & FL	WSI	ImageScope
Hamamatsu	NanoZoomer S60	BF & FL	WSI	–
Zeiss	AxioVision MosaiX	BF & FL	WSI	–
Olympus America	VS 110	BF & FL	WSI	–
Ventana/Roche	iScan	BF & FL	WSI	–
3DHistech	Pannoramic 250FLASH III	BF & FL	WSI	–
Huron Technologies	TISSUEscope 4000	BF & FL	WSI	–
MetaSystems	Metafer	BF & FL	WSI	–
MikroScan Technologies	MikroScan	BF & FL	WSI	–

BF, brightfield; FL, fluorescence; MSI, multispectral imaging; ROI, region of interest; WSI, whole slide imaging.

Another promising technology for mIHC, namely MultiOmyx™^21^ and tissue‐based cyclic immunofluorescence (t‐CyCIF),^26^ relies on dye cycling, in which repetitive cycles of labelling, image scanning, then fluorochrome bleaching and/or antibody stripping is performed. The level of multiplexing that can be obtained using such assays is much higher than of spectrally resolved assays, even with some technologies reporting acquisition of up to 60 markers per FFPE section.^26^ The advantages of using cyclic immunofluorescence are that it eliminates the need for expensive multispectral instruments, allowing multiplexed analyses across whole tissue sections of tumors using simple three‐colour whole slide scanning microscopes. In addition, such IHC methods can be performed using chromogenic dyes as well as fluorescence staining. However, this process can be very labour intensive because of the need to stain and image each biomarker sequentially,^21^, ^26^ resulting in prolonged turnaround time and potential changes of the tissue morphology and antigenicity because of repetitive exposure to the dye bleaching and/or antibody stripping conditions.^21^ Emerging antibody‐based imaging techniques,^27^, ^28^ such as imaging mass cytometry (IMC),^29^ multiplexed ion beam imaging (MIBI),^30^ Nanostring GeoMx^®^,^31^, ^32^ CODEX^®^™,^33^ InSituPlex^®^,^34^, ^35^ and MACSima™^36^, ^37^ can further produce omics‐like data through quantification of up to 40 markers. However, these technologies require longer measurement time, limiting the number of region of interests and size of the tissue that can be imaged (Table 5). Despite the challenges with limited scalability and throughput of each technique, multiplexed IHC methods enable simultaneous detection and co‐localisation analysis of multiple markers in intact tissue sections,^17^, ^18^, ^19^, ^42^ which has driven continued development in this field because of its potential for identification of clinically relevant biomarkers.

The utility of mfIHC relies on the ability to profile several markers simultaneously. However, the analysis of multiparametric information from mfIHC can be laborious to perform manually. Recent application of machine learning, deep learning and artificial intelligence in quantitative image analysis‐based multiplexing has enabled detailed representation of tumor tissue by generating quantifiable data that can precisely profile multiple different cell types in the TME.^43^, ^44^ Automated algorithms have further allowed researchers to segment cell nuclei and morphology of the tissue, providing broader information such as area or intensity of specific stains, and to compare different cell types in specific areas of interest in the tissue specimen (e.g. diseased and normal). This evolution of immune contexture of the tumor is also referred to as Immunoscore, which uses chromogenic IHC to measure the density of two lymphocyte populations on serial sections, in particular CD3^+^ and CD8^+^ T cells, both in the tumor centre and at the periphery of the tumor using digital pathology.^1^, ^7^ In an effort to promote the utilisation of Immunoscore in routine clinical settings, a worldwide Immunoscore consortium was initiated, with the support of the World Immunotherapy Council, of the Society for Immunotherapy of Cancer and several other societies.^45^ The consortium identified a strategy to demonstrate the feasibility, reproducibility, significance, robustness and prognostic power of Immunoscore^®^ assay in predicting stage II colon cancer patients with high risk of recurrence.^46^ Today, Immunoscore^®^ has already outperformed the standard tumor–node–metastasis (TNM) staging as a prognostic test for colorectal cancer.^3^, ^47^ Furthermore, to enhance the clinical utility and standardisation of this assay, an *in vitro* diagnostic (IVD) Immunoscore^®^ assay for clinical use has also been developed by the HalioDx immuno‐oncology company.^48^ Immunoscore^®^ is the first IVD immune scoring diagnostic test of the HalioDx pipeline, that is used by pathology laboratories leveraging advanced image analysis. Immunoscore^®^ is now validated for prognostic and predictive diagnosis of colon cancer. The presence of CD8^+^ T‐cell infiltrates has also been shown to have prognostic benefit in other cancer types, including melanoma, NSCC, RCC and bladder cancer.^49^, ^50^, ^51^, ^52^ There are also indications that other biomarkers may be clinically useful in enhancing Immunoscore^®^. For example, PD‐L1 is one such clinically relevant prognostic and predictive marker that has received FDA approval for its use with checkpoint inhibitors pembrolizumab and nivolumab.^8^, ^9^ However, not every patient with low levels of T‐cell infiltrates in their tumor rapidly progress, nor does every tumor with high PD‐L1 respond to anti‐PD‐L1 therapy. It is therefore important to explore Immunoscore^®^ with additional prognostic immune parameters on multiple cancer types.^1^, ^3^, ^45^, ^53^ Recent evidence has also shown that the presence of PD‐1 and Tim‐3 on CD8^+^ infiltrating T cells correlates with poor clinical outcome in renal cell carcinoma, indicating a possible exhausted phenotype.^52^, ^54^ Thus, the utilisation of multiparametric analysis to study the interactions and spatial relations between tumor and various immune cell phenotypes could further extend the prognostic and predictive implication of Immunoscore^®^, compared to CD8 and/or CD3 staining alone.

Integration of MSI and advanced digital image analysis technologies in multiplexed IHC samples has the potential to reveal co‐expression of immune molecules, pathway configurations and the spatial relationships between different immune and malignant cells within a particular tissue compartment.^18^, ^55^ Such quantitative spatial profiling of key immune‐ and tumor‐related pathways could improve the stratification of cancer patients for immunotherapy.^56^ It is therefore not surprising that there is a growing interest in combining IHC‐based multiplexed image analysis with artificial intelligence or machine learning to ensure reproducibility and robustness in interpreting tissue‐based information. Currently, there are several IHC‐based multiplexed image analysis software packages including Inform,^25^ Halo^57^ and Qupath^58^ that use machine learning to identify and characterise the multi‐parameter fluorescent and/or chromogenic profile of individual cells from digitised pathological slides. Such a combined multiplexing approach with advanced image analysis can offer flexibility and greater insight into disease pathogenesis by facilitating a systems biology approach.

## Future clinical applications of mfIHC

While advances in therapeutic strategies for cancer treatments have significantly improved survival in some patients, questions still remain as to why some patients do not respond. Since tumors are highly heterogeneous among individuals, further development of predictive markers may help maximise the clinical benefit and minimise the incidence of adverse events. In particular, the emerging success in immuno‐oncology requires delineation of complex crosstalk between immune‐ and tumor‐related pathways. Current efforts on novel biomarker candidates rely on identification and quantification of different immune cell populations, their spatial relationship, tumor mutation burden and immune gene signature.^59^, ^60^ As such recent technological advances combined with an explicit need to use multiple stains to characterise immune cells in different tissue compartments has seen an increased need for mfIHC in preclinical and clinical settings (Table 2). mfIHC has demonstrated that clinical correlation of high‐dimensional integrative analysis of the immune contexture – before and after therapy – is useful in identification of prognostic and predictive biomarkers in cancer patients.^56^ For example, expression of high PD‐1/PD‐L1 in patients with metastatic melanoma was associated with significantly improved progression‐free survival and overall survival, and these were also more likely to respond to anti‐PD‐1 monotherapy. Similarly, the densities of PD‐1 and PD‐L1 expressing cells in the TME of patients with Merkel cell carcinoma positively correlated with response to anti‐PD‐1 monotherapy.^61^ Tumor‐infiltrating lymphocytes have also been identified as a prognostic and predictive biomarker in breast cancer.^62^

**Table 2:** Applications of mfIHC in clinical studies

Tumor type	Makers studied	Multiplexed staining method	Imaging	Summary	Ref
Primary NSCLC	Pan‐Cytokeratin (AE1/AE3), PD‐L1, PD‐1, CD3, CD8 & CD68, granzyme B (GB), CD45 RO, Foxp3		Vectra ^®^ & InForm 2.1.0 Software (Akoya Biosciences)	T lymphocytes were predominantly concentrated in stromal compartment instead of epithelial compartment in NSCLC	63
Lung cancer (NSCLC)	AE1/AE3, PD‐L1, CD3, CD4, CD8, CD68, PD‐1, granzyme B, Foxp3, CD45RO, CD57	Opal 7‐Color fIHC Kit (Akoya Biosciences)	Vectra^®^ & InForm 2.1.0 Software (Akoya Biosciences)	Neoadjuvant chemotherapy activates immune response mechanism	66
Lung Cancer (NSCLC)	PD‐L1,CK, IDO‐1, B7‐H4, CD3, CD8, CD20	Sequential multiplexed IF (Alexa Dyes and fluorescence‐tyramide (Akoya Biosciences)	PM‐2000 image workstation & AQUA Analysis software (Genoptix,Inc.)	Localisation of Immune cells and their relationships with immunosuppressive markers in the tumor microenvironment	169
Lung Cancer (NSCLC)	DAPI,CK,CD3,CD8,CD20	Sequential multiplexed IF (Alexa Dyes and fluorescence‐tyramide (Akoya Biosciences)	PM‐2000 image workstation & AQUA Analysis software (Genoptix,Inc.)	Intra‐tumoral infiltration of cytotoxic T cells correlates with survival	50
Lung Cancer (NSCLC)	AE1/AE3, PD‐L1, CD3, CD4, CD8, CD68, PD1, granzyme B, Foxp3, CD45RO, CD57	Opal 7‐Color fIHC Kit (Akoya Biosciences)	Vectra^®^ & InForm 2.1.0 Software (Akoya Biosciences)	Epithelial lymphocytes and TMA’s associates with outcome	66
Lung Cancer	CD8, CD103, E‐cadherin, CD49a	Sequential staining Alexa Dyes	Axio Scan z1 (Zeiss)	Identification of prognostic composite biomarker	170
Lung Cancer	CD4, CD8, CD20, Foxp3, CD45RO	Opal 7‐Color fIHC Kit (Akoya Biosciences)	Vectra^®^ & InForm 2.1.0 Software (Akoya Biosciences)	Spatial distribution or relationship of lymphocytes subclasses associates with patient prognosis.	67
Breast Cancer	Cytokeratin, CD3, CD8, CD20	Opal 4‐Color fluorescent IHC kit and Alexa Dyes	PM‐2000 image workstation & AQUA Analysis software (Genoptix,Inc.)	Tumor‐infiltrating lymphocytes (TILs) predict response to neoadjuvant chemotherapy	62
Metastatic Colon cancer	PD‐L1, Epithelial cells (Pan‐cytokeratin), Helper T cell (CD3^+^CD8^+^Foxp3^+^), Cytotoxic T cell (CD3^+^CD8^+^), Regulatory T cell (CD3^+^CD8^+^Foxp3^+^), Antigen‐presenting cell (CD163)	Opal 7‐Color fIHC Kit (Akoya Biosciences)	Vectra^®^ & InForm 2.1.0 Software (Akoya Biosciences)	Prognostic role of cytotoxic T lymphocyte and PD‐L1 on APC as an immunosuppressive mechanism	64
Human gastric disease	PD‐L1, CD8, Foxp‐3	Opal 4‐Color fluorescent IHC kit (Akoya Biosciences)	Nikon C1 confocal microscope & ImageJ Software	Ratio of CD8^+^Foxp3^+^ and CD8^+^PD‐L1^+^ impacts tumor microenvironment	68
Glioblastoma multiforme	CD3, PD‐L1, Sox‐2	Opal 7‐Color fIHC Kit (Akoya Biosciences)	Vectra^®^ & InForm 2.1.0 Software (Akoya Biosciences)	Pre‐therapy microenvironment impact on response to T‐cell therapy	83
Metastatic melanoma	CD4, CD3, CD8, Foxp3, PD‐L1, CD20, CD68, CD11c, Sox10	Opal 7‐Color fIHC Kit (Akoya Biosciences)	Vectra^®^ & InForm 2.1.0 Software (Akoya Biosciences)	PD‐L1 expression on tumor and macrophages correlate with intra‐tumoral CD8 infiltration	69
Metastatic melanoma	PD‐1, PD‐L1	Alexa Fluor TSA kits (Invitrogen)	Halo^®^ (Indica Labs)	Therapeutic PD‐1 blockade induces responses by inhibiting adaptive immune resistance	75
Merkel cell carcinoma	CD8, CD4, CD20, PD‐1, PD‐L1, Foxp3, CD68 and neuron‐specific enolase (NSE, tumor cells)	Opal 7‐Color fIHC Kit (Akoya Biosciences)	Vectra^®^ &InForm 2.1.0 Software (Akoya Biosciences)	PD‐1 and PD‐L1 density correlates with response to anti‐PD‐1 therapy	61
Non‐small‐cell lung cancer	CD8, CD4, Foxp3, CD68	Opal 7‐Color fIHC Kit (Akoya Biosciences)	Vectra^®^ & InForm 2.1.0 Software (Akoya Biosciences)	Spatial interaction between tumor cells and regulatory T cells associated with poor survival	65
Colorectal cancer liver metastases	CD163, PD‐L1	Alexa Dyes	NDP NanoZoomer System (Hamamatsu Photonics) & VisioMorph Software (Visiopharm)	Spatial T‐cell heterogeneity in the invasive margin associates with improved survival	70
Pancreatic Cancer	Foxp3, CD4, Collagen‐I, CD8, Cytokeratin 8, αSMA, CD3	Opal 7‐Color fIHC Kit (Akoya Biosciences)	Vectra^®^ (Akoya Biosciences), Nuance Image analysis software (PerkinElmer) & ImageJ	Spatial distribution of intra‐tumoral T cells correlate with outcome	19
Breast cancer	Cytokeratin, CD3, CD8, CD20	Opal 4‐Color fluorescent IHC kit and Alexa dyes	AQUA Analysis software (Genoptix,Inc.)	Intra‐tumoral heterogeneity in TIL subpopulation in breast cancer	71
Breast cancer	Pan‐cytokeratin (AE1/AE3), PD‐L1	Alexa Dyes	AQUA Analysis software (Genoptix,Inc.)	PD‐L1 expression predicts response to neoadjuvant chemotherapy	80
Breast cancer	Pan‐cytokeratin (AE1/AE3), PD‐L1	Alexa Dyes	AQUA Analysis software (Genoptix,Inc.)	PD‐L1 expression decreases, while stromal TILs increase after neoadjuvant chemotherapy	79
Gastric Cancer	CD163, CD68, CD206, IRF8, PD‐L1, multi‐cytokeratin (NCL‐L AE1/AE3)	Opal 7‐Color fIHC Kit (Akoya Biosciences)	Vectra^®^ & InForm 2.1.0 Software (Akoya Biosciences)	Spatial distribution of TAMs associated with clinical outcome	73
Gastric Cancer	CD4, CD8, PD‐1, PD‐L1,Tim‐3, Foxp3	Opal 7‐Color fIHC Kit (Akoya Biosciences)	Vectra^®^ & InForm 2.2.0 Software (Akoya Biosciences)	Neoadjuvant chemotherapy increases the expression of checkpoint molecules and T‐cell infiltration	78
Metastatic Melanoma	PD‐1, Foxp3, Sox10, CD8, PD‐L1	Opal 7‐Color fIHC Kit (Akoya Biosciences)	Vectra^®^ & InForm 2.1.0 Software	Interspatial distribution of immune and tumor cells predicts response to anti‐PD‐1‐based therapy	74
Cervical cancer	CD8, CD3, Foxp3, T‐bet, Ki67	Opal 7‐Color fIHC Kit (Akoya Biosciences)	Confocal laser scanning TCS SP8 microscope (Leica), LAS AF Lite Software (Leica) & TissueStudio^®^ (Definiens)	Neoadjuvant cisplatin and paclitaxel induce tumor‐infiltrating T cells	81

The immune contexture is defined as the density, localisation and organisation of immune cell within solid tumors. Immune contexture analysis using mfIHC revealed high density of T lymphocytes concentrated in the stromal compartment but not in the epithelial compartment in NSCLC.^63^ Infiltration of intra‐tumoral CD3^+^ and CD8^+^ T cells in NSCLC was associated with better survival outcome, and the prognostic impact of CD8^+^ T‐cell infiltration was independent from age, tumor size, histology, and stage in multivariate analyses.^50^ Similarly, increased engagement of tumor epithelial cells with cytotoxic T lymphocytes (CD3^+^CD8^+^) in metastatic colon cancer was associated with improved overall survival. The abundance of infiltrating PD‐L1 expression on antigen‐presenting cells (APCs) in the TME indicates an immunosuppressive environment in these patients.^64^ By analysing tissue microarrays, an increased infiltration of CD8^+^ cytotoxic T cells was associated with improved patient outcome, and increased infiltration of regulatory T cells into core regions was identified as an independent marker of poor patient outcome in NSCLC.^65^

Studies have now reported that not just one cell type, but the relationship between different immune cells in different tumor compartments have prognostic benefit that can impact on patient survival. For example, after neoadjuvant chemotherapy, higher levels of epithelial lymphocytes (CD3^+^CD4^+^) and epithelial and stromal tumor‐associated macrophages (CD68^+^) were associated with better outcome in patients with NSCLC.^66^ A higher effector CD8^+^ T‐cell/regulatory T‐cell ratio in the tumor compartment, and a higher intra‐tumoral/stromal ratio of CD8^+^ effector cell infiltration correlated with better overall survival in patients with NSCLC.^67^ Furthermore, the ratio of cytotoxic T cells to regulatory T cells (CD8^+^:Foxp3^+^), and cytotoxic T cells to PD‐L1 (CD8^+^:PD‐L1^+^) were also found to be suppressed in the microenvironment of gastric cancer tissues compared to those of normal adjacent gastric tissues.^68^ In metastatic melanoma, PD‐L1^+^ expression on both melanoma cells and macrophages was shown to correlate with high levels of intra‐tumoral CD8^+^ cells but not with intra‐tumoral CD4^+^ Tregs.^69^ Similarly, the intercellular interactions between tumor cells and regulatory T cells in non‐small‐cell lung cancer were associated with poor survival, while the interactions between CD8^+^ T cells and regulatory T cells correlated with improved survival.^65^

The application of mfIHC in immune contexture analysis has become increasingly useful for the performance of spatial distribution analysis of immune and tumor cells in various cancers, including colorectal cancer, pancreatic cancer and breast cancer.^19^, ^70^, ^71^ While the relative distribution of immune cells in different compartments of tumors is known to influence disease progression and response to immunotherapy, the spatial interactions between immune and tumor cells can greatly impact the overall tumor ecosystem and have significant influence on tumor progression and therapy responses.^7^, ^72^ Several studies have now demonstrated the association between spatial distribution of immune cells and prognosis in various cancers.^65^, ^70^ In patients with liver metastasis from colorectal cancer, the distribution of T cells in close proximity (≤ 10 µm) to the tumor periphery has been associated with improved overall survival.^70^ In gastric cancer, the clinical outcome was associated with high number of tumor‐associated macrophages (CD68^+^CD163^+^) and their proximity to tumor cells.^73^ Similarly, the spatial distribution of cytotoxic T cells in proximity to pancreatic cancer cells correlated with increased overall survival.^19^ The intercellular spatial distribution of immune cells within 20 µm of melanoma cells in pre‐treatment metastatic melanoma specimens was significantly associated with response to anti‐PD‐1 monotherapy and progression‐free survival.^74^ Furthermore, the proximity of PD‐1‐expressing cells and PD‐L1 in pre‐treatment metastatic melanoma specimen correlated with a positive response to pembrolizumab.^75^ The prognostic potential of multiplexed IHC technology is not just limited to immunotherapy but is also applicable to conventional therapies such as chemotherapy. For example, several studies have shown that neoadjuvant chemotherapy, whatever the regimen, can alter the TME, thereby possibly making it more favorable for immunotherapy.^62^, ^66^, ^76^, ^77^, ^78^, ^79^, ^80^, ^81^, ^82^ Thus, the quantitative spatial profiling of immune markers via multiplexed immunofluorescence may be a useful tool for treatment selection and biomarker identification for single or combination therapy.

Multiplex fluorescence IHC has proved to be useful to delineate the immune‐tumor pathways and their spatial relations in stratification of patients for immunotherapy. For example, we recently used quantitative fluorescence multiplexing imaging techniques to demonstrate that the cellular makeup in pre‐treatment tissue from recurring GBM patients can predict the long‐term response following autologous CMV‐specific T‐cell therapy. While the long‐term survivors had significantly reduced number of CD3^+^ T cells in comparison with short‐term survivors, a proportion of short‐term survivors displayed higher PD‐L1 expression. These data suggest that combining T‐cell therapy with PD‐1/PD‐L1 blockade may improve overall survival of GBM patients.^83^ Therefore, quantitative fluorescence multiplexed IHC technology as a platform for diagnostic and prognostic biomarker identification is poised to revolutionise traditional pathological interpretation.

## Challenges in advancing the utility of mfIHC in immuno‐oncology

As the field of immune oncology continues to grow, the need for standardised multiplexed IHC for relevant tissue biomarkers, together with an accurate and reproducible image analysis pipeline, is likely to provide increased support for future clinical decisions. Advances in mfIHC technology, digitisation and automated image analysis have the potential to provide robust and reproducible multiplexed IHC data for personalised treatment of patients with immunotherapy. Despite these technological advancements, there are several factors that impede the implementation of mfIHC‐based quantitative digital pathology in clinical settings. Overcoming current limitations and providing a unified workflow will be essential for developing full, widespread, clinical benefit of this technology. These limitations can be roughly categorised as follows: tissue handling and processing, tissue staining, image acquisition and digital pathology, quantitative image analysis and centralised workflow.

### Tissue handling and processing

The reproducibility of IHC data can be greatly affected by sample handling, which begins with tissue collection (at autopsy or biopsy) until a section is ready to be stained.^84^, ^85^ Prior to fixation, the ischaemic time of resected tissue is crucial to prevent tissue from degradation and autolysis.^86^, ^87^ The volume of fixative and types and length of fixation can also affect the epitopes which can result in differences in staining patterns. Generally, 10% neutral buffered formalin is used as a standard fixative in most hospitals; however, the choice of fixative is dependent on the downstream technique that will be applied to the tissue.^85^, ^88^ The optimal time for fixation is 24 h which can vary depending on the thickness of the tissue and epitope to be evaluated.^89^ Multiple additional factors that occur through processing to sectioning of the tissue can incur additional variation. For example, the duration of storage, temperature of storage, sample orientation, thickness of the section, environmental exposure, oxidation and the type of antigen being investigated can all influence the immunoreactivity and antigenicity.^85^, ^90^, ^91^ Consistent tissue thickness using a validated sample preparation protocol is critical to maintain morphology and ensure reproducible IHC data. Thicker sections give darker staining with lower resolution while thin sections offer lighter staining with enhanced resolution. A thickness of 3–4 µm tissue sections is considered ideal for downstream single or multiplexed IHC.^88^, ^92^ For IHC that requires simultaneous staining of multiple markers, the sections must be transferred to positively charged slides and allowed to dry overnight before staining. The unstained sections are subject to oxidation and environmental temperature, which inversely affects antigenicity (antigen instability).^93^, ^94^, ^95^ In contrast, paraffin blocks are resistant to antigen degradation and can be stored for several decades.^96^, ^97^ Thus, for short term, the unstained sections may be stored at 4°C although staining the sections immediately after sectioning is generally recommended.^98^, ^99^

### Tissue staining

Staining of FFPE tissue sections can introduce variability during scoring.^85^, ^88^, ^100^ For example, the method of staining (direct versus indirect), target protein being evaluated, the time and temperature of staining, antibody type (monoclonal versus polyclonal), dilution, origin of species and vendors, dilution and type of detection reagents, method of antigen retrieval and epitope retrieval buffer, blocking solution and the type of counterstain used can all affect the score data.^101^ The increasing use of fluorescence‐based multiplexed IHC staining in the study of immune contexture introduces additional variables, such as type of staining techniques (sequential versus simultaneous), the localisation of the marker under evaluation, antibody cross‐reactivity, antigen retrieval method, spectral overlap between multiple labels, photo bleaching, tissue autofluorescence and signal quenching can all affect the fluorescence readout.^20^, ^102^, ^103^

Several different approaches to fluorescence‐based multiplex IHC have been developed, which can be categorised as sequential or simultaneous depending on the choice of staining.^104^ Sequential fluorescence mIHC involves labelling the tissue with one or two antibodies at a time, where the antibodies or antibody complex is stripped or the fluorophores are quenched between each cycle of staining.^21^, ^104^ Sequential staining using antibody stripping offers simultaneous detection of multiple markers.^104^ In dye cycling‐based approaches, images of the slide are acquired between each cycle of staining, and then, the images from each cycle are co‐registered to generate the multiplex image.^20^, ^21^, ^26^ Such sequential staining methods can cause issues such as cross‐reactivity between different antibodies, signal cross‐reactivity because of incomplete elution of antibodies or quenching of fluorescence signal, and may also lead to disruption of epitopes or tissue integrity because of repeated cycle of heating or treatment with harsh chemical bleaching to sequentially label multiple antigens. The images generated from each cycle of staining must also be registered accurately to ensure the integrity of the data.^21^, ^26^

Simultaneous fluorescence mIHC involves using multiple antibodies (conjugated or unconjugated) to label different antigens on the same slide at the same time. This type of staining is more time efficient, is not influenced by sequential rounds of imaging, and causes less tissue damage, but is limited to the number of fluorophores that can be resolved from each other during image acquisition. This method may incur artefacts because of spectral overlap of the dyes, also known as fluorescence bleed through, which can create difficulties in separating discrete fluorescence signals and complicate the evaluation of co‐localisation experiments.^18^, ^105^ The degree of spectral overlap in a particular experiment is dependent the choice of fluorochromes and the antibodies used.^106^ Some dyes have wide emission spectra which can contribute strongly to spectral overlap, while others emit over a narrow range of wavelengths and usually have less spectral overlap depending on the brightness of the fluorophore and the fluorophore combination used.^105^ Thus, the brightest fluorophores should generally be used to label antigens with the least abundant expression and dimmer fluorophores should be used to label the most abundant proteins. Suboptimal design of the multiplexed panel in fluorescence mIHC can therefore negatively affect the quality of data because the signal is too dim and cannot be imaged effectively or too bright causing excessive bleed through. The sequence of antibody staining in the panel should be validated and not changed because the microwave/heat‐induced epitope retrieval step that is performed between each antibody, and the duration of heating can affect the stability and quality of staining for all antibodies in a multiplex panel. It is therefore not feasible to simply merge a validated single‐plex fluorescence IHC protocol into a multiplexed methodology. A single change in the antibody, reagent, tissue type or the antibody order in multiplexed panel can require extensive rearrangement of fluorochrome–antibody combinations and optimisation of the protocol for optimum or acceptable performance. To preserve the quality of data and minimise the panel development time, it is generally advisable to develop a standardised panel with a fixed set of markers that meets a particular purpose.

One of the biggest challenges in quantification of fluorescence signal in FFPE tissue is tissue autofluorescence.^103^, ^107^, ^108^, ^109^ Each cell has its own intrinsic level of fluorescence which generally emits over a broad spectrum and can impact almost every fluorescent stain.^109^ Thus, a fluorescent staining protocol designed for one type of tissue may not be suitable for a different tissue. It is therefore advisable that an antibody panel for staining is optimised using the same tissue type and even from the same tissue block if possible. The signal from each antibody staining should be validated in a single‐plex IHC before combining the makers in a multiplexed panel. It is also essential to include appropriate positive and negative controls to validate the sensitivity, specificity and reproducibility of the IHC protocol for a given antibody.^110^, ^111^, ^112^ This is particularly important when the markers are being quantitatively assessed for staining intensity. Positive controls are tissues or samples containing the marker(s) that have areas which gives different levels of staining when visualised by a stain. The use of a negative control is essential to check for nonspecific staining or artefacts (false‐positive result) resulting from the primary antibody. Appropriate negative control slides include an isotype control or a cell line or tissue that does not express the protein of interest. A secondary alone control is also required to ensure that the secondary antibody does not exhibit nonspecific binding. However, it does not provide information regarding specificity of staining with the primary antibody. Importantly, the quality of the staining pattern as per localisation of markers such as extracellular, intracellular or subcellular distribution (nucleus, cytoplasm and membrane) must be validated with the assistance of trained pathologist to prevent quantification error. Each step in staining process can potentially be a source of variation in score data. This also includes the type of staining method used – manual versus automated staining. For instance, manual staining tends to have more opportunities for errors and variations compared to automated systems. Staining of a large volume of slides generally requires multiple runs (‘batches’), and each batch of a run has the potential to produce differences in the quality of staining. Thus, randomisation of the slides can prevent biasing the data and use of an appropriate control slide can help ensure the sensitivity of staining as well as the reproducibility of data.

### Image acquisition and digital pathology

Histopathological evaluation of tumor tissue has been traditionally performed by pathologists using a standard microscope and a semi‐quantitative scoring system.^84^, ^113^ Recent advances in technology have allowed researchers and clinicians to take whole slide image of IHC slides which may improve the safety, quality and efficiency in diagnostic workflow.^114^ Whole slide imaging (WSI) refers to digitisation of entire specimen into a single digital slide that allows for interpretation and management of specimen in an image‐based environment.^115^, ^116^ These digital images can be accessed remotely to facilitate telepathology (the practice of pathology from a distance), outsourcing and consultation for routine cases in areas that requires pathologist’s expertise, including developing countries.^117^, ^118^ In addition, WSI enables the generation of whole slide digital tissue banking which can be archived for research, molecular testing, medico‐legal and forensic purposes.^119^, ^120^, ^121^

Whole slide imaging can be categorised into three types, brightfield, fluorescent and MSI, depending on the type of scanner used.^114^, ^122^ Brightfield scanners digitise chromogen‐based IHC and are most commonly used in clinical practice. Fluorescence scanners capture fluorescently labelled slides using a monochrome camera attached to a microscope that is equipped with specific filters and mirrors to separate the multiple fluorescent signals. MSI captures images at a discrete spectral intervals and can be used for both brightfield and fluorescent imaging.^122^ Capturing MSI of WSI takes longer and generates larger files than non‐MSI slide scanners but there has been continued development in this area because of its potential in clinical application, and MSI technologies have proven to be useful in both preclinical research and clinical pathology.^123^

WSI scanners that allow for high‐speed digitisation of fluorescence IHC glass slides are available from many different vendors.^124^ Some well‐known fluorescent WSI scanners include Aperio FL, Vectra^®^, Vectra^®^ Polaris™ and Hamamatsu NanoZoomer. Different scanner models vary in terms of their features and functionality,^125^ and the selection of WSI scanner depends on its intended use such as the type of specimen being scanned (FFPE, frozen sections), type of stain (chromogenic or fluorescent), number of markers being evaluated (single‐plex or multiplex), usage in a clinical or non‐clinical laboratory, type of glass slide being scanned, the downstream companion software and laboratory information system (LIS) required to manage and support the clinical workflow as well as the cost associated with the purchase and maintenance of the WSI scanners.^126^, ^127^, ^128^

Most WSI scanners for fluorescence IHC comprise a software‐driven, robotically controlled microscope with high‐quality objective lenses, high‐quality monochrome cameras, and multiple filter cubes for single‐plex or multiplex imaging.^119^, ^129^ It is therefore important to consider the light source (brightfield versus fluorescence), scan magnification, scan time, slide holder capacity, hardware robustness, stitching algorithms, scan failure rate, *z*‐stacking for 3‐D reconstruction, image resolution, image quality, file compression methods, formats and file size when selecting a slide scanner for digitisation of FFPE slides.^128^, ^130^ Histopathology specimens are relatively easy and quick to scan compared to cytology specimens because they have smooth topology and small depth variations.^131^, ^132^ Scanning of cytology slides may require a multi‐planar scanner with *z*‐stack capabilities,^133^ which is not described in depth here. Ideally, a routine surgical pathology specimen can be scanned at a low magnification (e.g. 20×); however, small objects such as microorganisms can only be identified with high magnification (e.g. 40× or greater) which offers better resolution.^134^ The scan time of FFPE slides can increase with higher magnification, larger tissue size, number of field of views or tiles, number of channels used in multiplexed stains, tissue section density, low signal strength and low signal‐to‐noise ratio.

Multispectral fluorescent WSI requires capturing and storing images at various wavelengths that are later processed to account for bleed through of the individual dyes and autofluorescence. The number of wavelengths captured increases the overall scan time and file size. Technical limitations in scanning can lead to imaging artefacts, such as lower image resolution, heterogeneous staining intensities and patterns, poorly focussed scans, improper stitching of lines or tiles, or overlapping signals from multiplexed spectra, all of which directly affect data accuracy and reproducibility.^130^, ^135^ While the optical resolution depends on the magnification and numerical aperture of the objective lens, it is also important to note that the digital resolution of the image may vary based on the detector/camera in the scanner, and the quality of the viewed image will depend on the monitor where the images are displayed.^136^ Establishment of a sound digital pathology workflow in a clinical or non‐clinical laboratory therefore requires additional considerations above the traditional histology workflow.^127^ These include the need for adequate staffing, proper training of personnel and pathologists, setting up pathologists’ workstations, additional quality control steps, availability and timely maintenance of equipment (e.g. scanner), adequate information technology infrastructure (e.g. server and computer), integration with LIS, standard operating procedures and guidelines for managed workflow.^125^, ^135^

Digital pathology has been successfully implemented around the world for education, clinical pathology conferences and research purposes.^75^, ^119^, ^137^, ^138^, ^139^ WSI systems such as the Omnyx™ Integrated Digital Pathology IDP by GE healthcare have been approved by Health Canada for all purposes in routine pathology such as creating, managing, storing, annotating, measuring, viewing digital whole‐slide images, and primary diagnosis.^140^, ^141^ The US Food and Drug Administration has also approved the Philips IntelliSite Pathology Solutions for primary diagnosis using surgical pathology slides, which has led to further interest in development and clinical adoption of digital pathology for diagnostic purposes.^142^ The widespread clinical implementation of Immunoscore^®^ requires an optimal biomarker study to be hypothesis driven, reproducible, with prognostic and/or predictive power and cost‐effective.^143^ Digital tools have the potential to facilitate pathology workflows for assessment of established immune biomarkers and enable deeper characterisation of TME. While some clinical centres utilise slide scanning to digitise histopathology samples,^144^ there are only a few centres that utilise complete digital pathology for routine histopathology.^145^, ^146^

Digital pathology systems that support multiplexing have not yet been adopted in clinical pathology laboratories; however, they are widely used in early discovery and clinical studies. Although the widespread adoption of mfIHC in the clinic may still be futuristic, contract research organisations (CROs) are currently offering high‐quality image acquisition and analysis based on mfIHC to clinic and research which may be helpful in patient management (Table 3). Because of the potential of mfIHC for use in the clinic, many pharmaceutical and biotechnology companies have made major investments in digital pathology, which includes automation in histology techniques (sample storage, tissue sectioning, staining), slide scanners and image analysis software. Thus, as a platform for diagnostic and prognostic biomarker identification, digital pathology is poised to revolutionise traditional pathologic interpretation, and implementing the regulatory guidelines that governs its use will need to be revisited.^147^

**Table 3:** Contract Research Organisations providing multiplexed IHC and IF services

Contract Research Organisations	Histology Services	Image acquisition and scanning platforms	Image analysis platforms	Links
Md Biosciences	Tissue Processing, IHC/ISH/IF, mIHC/mfIHC	Aperio VERSA (Leica), Ventana DP‐200 BF Imager (Roche)	Indica Labs^®^ HALO^®^, Visiopharm Aperio ImageScope, FIJI/ImageJ	https://www.mdbiosciences.com/
Cell IDx	Tissue Processing, IHC/IF, mIHC/mfIHC	Aperio Versa Scanner (Leica)	Visiopharm, Leica analytical software	https://cellidx.com/
HalioDx	Tissue Processing, IHC/mIHC	NanoZoomer Scanner (Hamamatsu)	Immunoscore^®^ Analyzer, In‐house Software	https://www.haliodx.com/
Visikol	Tissue Processing, IHC/IF, IMC‐mIHC/mfIHC	ImageXpress^®^ Confocal (Moleculardevices), CellInsight CX7 LZR HCS platform (Thermo Fisher), Aperio VERSA 8 (Leica), CyTOF^®^ Technology (Fluidigm)	In‐house Software, Python	https://visikol.com/
SironaDx	Tissue Processing, IHC/ISH/IF, mIHC/IMC‐mIHC/mfIHC	CyTOF^®^ Technology (Fluidigm), CODEX (Akoya Biosciences), Vectra^®^ Polaris^®^ (Akoya Biosciences)	In‐house Software, Inform, Indica Labs^®^ HALO^®^	https://sironadx.com/
Akoya Biosciences	Tissue Processing, IHC/ISH/FISH/IF, mIHC/mfIHC	Vectra ^®^ Vectra^®^ Polaris™ (Akoya Biosciences)	Inform, Indica Labs^®^ HALO^®^	https://www.akoyabio.com/
Aquila Biomedical	Tissue Processing, IHC/ISH/IF, mIHC/mfIHC	Vectra^®^ Polaris™ (Akoya Biosciences), Thunder (Leica)	Indica Labs^®^ HALO^®^, Visiopharm, Oncotopix^®^, Definiens	https://aquila-bm.com/
PhenoPath	Tissue Processing, IHC/ISH/FISH/IF, IMC‐mIHC and mfIHC	Aperio whole slide scanner (Leica), Nuance (PerkinElmer)	Inform	http://phenopath.com/
Ultivue	Tissue Processing, IHC/ISH/IF, mIHC and mfIHC	SLIDEVIEW™ VS200 (Olympus), Zeiss Axio Scan.z1 (Zeiss)	UltiStacker™ Software, Indica Labs^®^ HALO^®^	https://ultivue.com/

FISH, fluorescent *in situ* hybridisation; IF, immunofluorescence; IHC, immunohistochemistry; ISH, *in situ* hybridisation; mfIHC, multiplexed fluorescence immunohistochemistry; mIHC, multiplexed immunohistochemistry.

### Quantitative digital image analysis and deep learning

Multiplexed IHC allows simultaneous detection and co‐localisation analysis of multiple markers *in situ* in the intact spatial context of tissues.^17^, ^18^, ^19^, ^21^, ^62^, ^89^ The emergence of digital pathology, and its application in translational science, has allowed researchers to see cancer differently. Whole slide digital images of tissue sections contain information that includes colour, tissue morphology, cell morphology and complex cell phenotypes. Through technology such as MSI‐based multiplexing, it is now possible to explore complex phenotypes and their interaction in the TME.^148^ Quantitating the number of cells that display a particular phenotype in a specific context of tumor tissue is important to explore immune evasion and predict and track response to therapy.^138^ Image analysis in pathology was primarily done through visual assessment of marker of IHC or IF samples. However, the standard pathological assessment of tissue includes several inter‐ and intra‐observer variation, longer duration for assessment, as well as difficulty in distinguishing co‐localisation markers when several fluorophores are being used simultaneously in one sample.^107^, ^149^, ^150^ Thus, to overcome many of these challenges, number of image analysis methods are being developed which can provide quantitative, per‐cell measurements from multi‐labelled IHC or IF samples.^151^ The basic principle of automated histopathologic image analysis generally involves three key steps: unmixing of fluorochromes to separate markers, automated identification of morphologic region (regions) in the tissue section, and cellular segmentation to enable quantification of intensity of one or more markers in a cell or subcellular compartment (e.g. nucleus, cytoplasm, membrane). These automated image analysis workflows are being increasingly used in diagnostic and investigative pathology.^114^, ^152^, ^153^

There are already a growing number of image acquisition platforms in the market that combine image analysis with automated slide scanning to support fluorescence multiplexed staining. These include the Akoya Biosciences Vectra^®^ and Vectra^®^ Polaris™,^24^ TissueGnostics/TissueFAXS,^154^ and Leica Biosystems/Aperio FL,^155^ all of which can scan slides affixed to whole tissue or tissue microarray slices and provide some level of image analysis. Higher level of multiplexing for multivariate biomarker analysis requires spectral unmixing to separate spectral overlap. Fortunately, some of the integrated scanning and analysis instruments use multispectral cameras that support unmixing for multiplexing modalities. Several stand‐alone, open or commercial fluorescence image analysis software packages are also available to evaluate digitised fluorescence multiplexed slides from various instruments. These include Halo,^57^ iGen, Cell Profiler, AQUA Analysis, QuPath,^58^ Icy, ImageJ and MATLAB (Table 4). These software packages perhaps integrate machine learning and deep learning algorithms that have contributed immensely towards advancing the potential of digital pathology. Software packages that integrate such algorithms are often developed for specific purposes such as *in situ* hybridisation (chromogenic or fluorescent), nuclear/cytoplasm/membrane biomarker identification, co‐localisation studies and spatial distribution analysis. There are now an increasing number of laboratories that are incorporating IHC image analysis software into their workflow for single‐cell‐based quantitative pathology. Each software package varies in terms of their flexibility, complexity, application and its ability to work on whole slides versus tiled (static) images, brightfield versus fluorescent images and different file formats.^84^, ^156^ Furthermore, image analysis of multiplexed IHC assays that incorporate greater than six markers could require assessment of each marker set in sequential process. Such staining techniques will require an image registration feature that allows integration of images after each round of staining,^104^ unlike other methodologies where the image is acquired at the end of the staining process. Although such techniques require longer time to stain, the multi‐stacked tissue generated from such staining methods is useful to perform multivariate biomarker analysis. Collectively, the image analysis workflow should include an automated tissue and cell segmentation feature, and integrate spatial co‐localisation of cell and distribution analysis such as co‐localisation of cells, distance between different cell populations or distance from the tumor region.

**Table 4:** Fluorescence image analysis software packages for quantitative multiplexed IHC

Algorithm type	Company	Software	Spectral unmixing	Tissue segmentation	Data representation	Batch mode	Image registration	Availability	Ref.
Unsupervised	Akoya Biosciences	InForm	Unmix	Tissue and cell segmentation, Automated Cell Phenotyping, Co‐localisation	Density Raw Data	✓	X	Licensed	18, 171
	TissueGnostics	StrataQuest	Unmix	Tissue and cell segmentation, Cell phenotyping	Density Raw Data, Dot Plots	✓	✓	Licensed	172
	Indica Labs	HALO	–	Tissue and cell segmentation, Cell phenotyping, Immune cell proximity, Spatial Analysis	Density Raw Data, Spatial Plot, Histogram	✓	✓	Licensed	57
	CompuCyte	iGen	–	Nucleus segmentation or Phantom contouring, Measuring associated signals	Density Raw Data Dot Plot, Histogram	✓	Unknown	Licensed	173
	Cell Profiler	Cell Profiler	–	Tissue and cell Segmentation, Cell phenotyping, Co‐localisation	Density Raw Data Dot Plot, Histogram	✓	X	Open	174, 175
	Leica Biosystems	Aperio IF	–	Pixel based quantification, object identification, Area quantification	Density Raw Data Histogram	X	**X**	Licensed	155
	Genoptix	AQUA Analysis	–	Signal intensity per unit area and per layer	Density Raw Data	X	✓	Licensed	176
	QuPath	QuPath	–	Tissue and cell Segmentation	Density Raw Data, Histogram	X	X	Open	58
	Icy	Icy	–	Object identification, Spatial Analysis	Density Raw Data	X	X	Open	177
Supervised	NIH	ImageJ	Unmix	Colour‐based, user interactive segmentation	Density Raw Data	X	✓	Open	178
	MATLAB	MATLAB	–	Tissue and cell Segmentation, Cell Phenotyping	Density Raw Data, Spatial Plot, Histogram	✓	Unknown	Licensed	179

Based on the level of training required, image analysis software can be classified into two groups: ‘Unsupervised’ and ‘Supervised’.^85^, ^157^ Unsupervised software packages enable researchers to score image data without the need for computational skills. These packages require minimal user training but offer less flexibility to perform higher order analyses for complex investigative studies. In contrast, supervised software packages allow users to perform complex analysis but require upfront training and more user input, which can be tedious to use in diagnostic settings.^85^ Supervised and unsupervised analyses of cell populations are common place in immunology and clinical diagnosis. For example, standard gating of cell populations during flow cytometry requires user input to accurately quantitate cell populations identified using multi‐parameter staining. Unsupervised learning such as tSNE is also commonly used by flow cytometry analysts to identify potential biomarkers.

**Figure 2:**
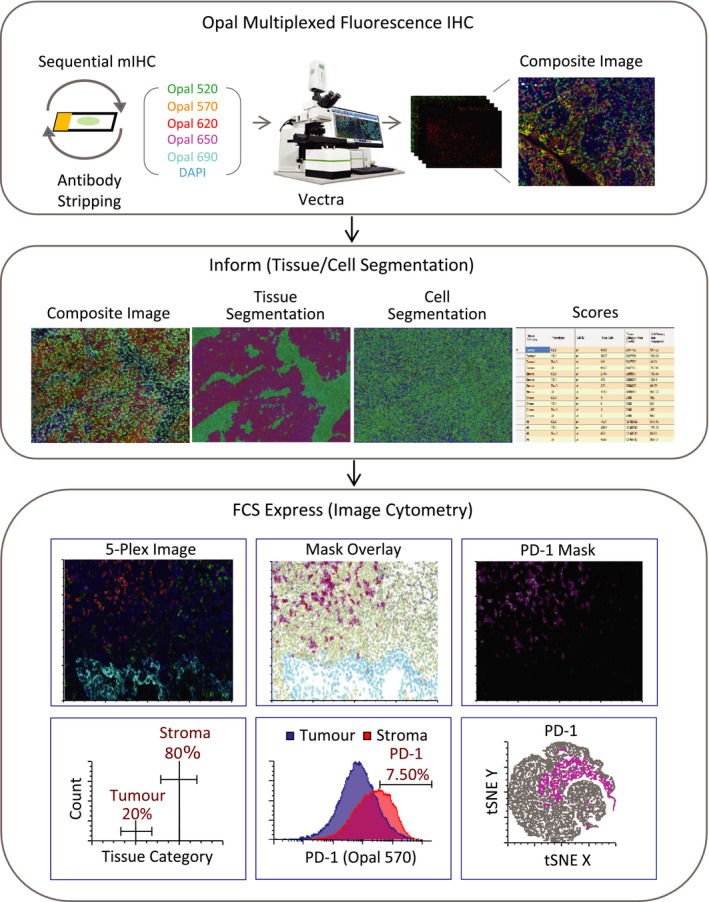
Work flow and general framework for multiplexed image analysis using Vectra quantitative digital pathology system and FCS express (flow and imaging). Typical steps involved in the fluorescence image analysis: Opal multiplexed fluorescence IHC, Vectra image scanning, Inform (Unmixing, Tissue Segmentation, Cell Segmentation, score), FCS export and image cytometry [Top: Unmixed image (left), Picture plot (middle) showing mask overlays on PD‐1‐positive cells (magenta), stromal cells (green) and tumor cells (cyan), Picture plot highlighting PD‐1‐positive cells (right); Bottom: Histogram showing tissue category region gated for cells in tumor and stroma (left), Histogram overlays (middle) of PD‐1 expression on tumor and stroma quantified from merged image data (12 fields of view from same slide), tSNE map showing PD‐1 expression highlighted in magenta (right)].

In collaboration with De Novo Software, we have integrated the ability to use FCS Express 6 Image Cytometry software to open and analyse fluorescence multiplexed images that were acquired using the Vectra^®^ automated quantitative digital pathology imaging platform. This integration allows for efficient exploration and quantification of large, multi‐parameter single‐cell image datasets while allowing access to the original images for visualisation. This method is useful to analyse cellular phenotypes and their relationships within spatial context of the TME in intact FFPE tissue sections. A typical data analysis pipeline for immune contexture analysis that utilises image cytometry software like FCS Express 6 for multiplexed digital images from the Vectra automated quantitative digital pathology platform is presented in Figure 2. This new approach allows us to explore multiplexed fluorescence IHC data to find biomarkers in an unsupervised manner. Since flow cytometry is commonly used in research and clinical settings, this type of analysis can provide image data in a manner that is readily transferable between research and diagnostic laboratories. The data can also be further inspected at the level of the nucleus, cytoplasm, membrane or total fluorescence, which provides an additional benefit over flow cytometry data which generally measures total cellular fluorescence and requires further staining protocols to obtain data on subcellular location (Figure 2). The education on the integration and analysis of mfIHC using Vectra and FCS express can be obtained through an online webinar portal in De Novo Software.^158^ FCS express also includes several other data visualisation tools (histogram, dot plots, density plots), reporting tools (bar chart, pie chart, line graphs), statistics (*P*‐values) and high‐dimensional data reduction tools such as tSNE, SPADE and *K*‐means, which can be applicable for high‐throughput analysis of multispectral data generated from multiplexed IHC platform.

In the setting of pathology, deep learning methods have been widely used in H&E‐ or IHC‐stained whole slide images for detection of metastatic loci, tumor classification and prediction of gene mutations.^159^, ^160^ IHC algorithms associated with different imaging platforms have received US FDA clearance as diagnostic tools.^161^ So far, five predictive IHC‐based biomarkers for immune contexture have been approved by the FDA as companion diagnostics including PD‐L1 for non‐small‐cell lung cancer, gastric or gastroesophageal junction, ALK for non‐small‐cell lung cancer, EGFR for colorectal cancer, CD117 for gastrointestinal stromal tumor and Her‐2 for breast cancer and gastric cancer. Roche has developed a companion algorithm image analysis software for Her2, which is the only IHC‐based marker that has received clearance from FDA for semi‐quantitative measurement of Her2 (4B5) in breast cancer patients.

Several commercial image analysis software packages that integrate the workflow exist for non‐clinical settings. The workflow of such commercial systems still carries limitations and challenges before its routine implementation in clinical decision‐making. Thereby, in addition to the issues explained above, the challenges in image analysis workflow also need optimisation and standardisation for its effective integration and translation into clinic.

Some of the most common image analysis artefacts includes cell segmentation errors (e.g. over segmentation or under segmentation of nuclei) and tissue classification errors (e.g. classifying tumor as stroma and vice versa). Therefore, an appropriate quality control measure must be included to assess potential artefacts throughout the IHC and image analysis workflow. It is important that pathologists are involved in reviewing the quality control step of the image analysis workflow that will otherwise influence the results.

In the area of image data analysis, there has been much advancement in tissue and cell segmentation; however, the current software packages still have limitations in phenotyping cells of different sizes and morphologies, distinguishing cells in close proximity, and processing spatial distribution analysis. Different software packages use tools such as deep learning or threshold‐based methods (like pixel intensity) to identify and label objects. These tools are effective for identification of single‐cell type; however, it is challenging to perform co‐expression and multivariate biomarker analysis.

One of the biggest challenges in translating tissue‐based diagnostic biomarkers in clinical decision is to determine a clear threshold for patient stratification based on expression of single or multiple markers. Since the staining results of all parameters are provided in a continuous variable, appropriate cut‐off values for each marker should be designated depending on the distribution of staining intensity, absolute number or both. The ‘optimal’ cut‐off point is defined as that threshold value of the continuous covariate distribution, which best separates low‐ and high‐risk patients with response to outcome.^162^, ^163^ Pathologists often use packages such as SPSS (IBM SPSS Inc.), GraphPad Prism (GraphPad Software Inc.) or Winstat (R.Fitch Software) in order to correlate biomarkers with outcome or survival data. The cut‐off values are often chosen using simple approaches like mean, median, quantile distribution of the biomarkers or adjusted manually. However, because of the fact that many factors can affect the quality of multiplexed staining and their subsequent IHC score, it is necessary to use methods that support distribution‐based cut‐off optimisation or cut‐off optimisation in context of a survival variable. Determining the best cut‐off point is often a compromise because of the staining intensities differences between center versus edge/periphery of the tissue and tumor versus stromal region. There are algorithms such as OptimalCutpoint^164^ and maxstat^165^ that predicts the cut point in a continuous variable; however, those programs are not user friendly and require programming knowledge. Other stand‐alone programs such as Cutoff Finder,^166^ X‐tile^167^ and Evaluate Cutpoint^168^ are also being used for cut‐off point determination of a continuous variable, but each has its own limitation in terms of selection of statistical algorithms. Therefore, a consensus will be required to set thresholds for stratification at a per tissue level in order to improve the quality of biomarker studies. With the growth in cancer immunotherapy, the clinical importance of quantitative and spatial characterisation of TME for diagnostic/prognostic biomarker studies is likely to increase. While deep learning methods have had marked impact in digital pathology, further improvement is required to expand its application in automated image analysis workflow.

### Centralised workflow

Automated image analysis software generates large volumes of high‐quality labelled data with raw information about the individual fluorescence profile in each cellular compartment. These data become more complicated to analyse when multiple samples are batch processed and compared simultaneously. Thus, downstream analytics software such as Excel, Spotfire, R studio, GraphPad Prism, or FCS express is required to inspect, analyse or graphically represent these data and also to perform standard set of statistics which can be associated with the clinical‐pathological parameters. Quantitative imaging informatics also requires tools that can allow simultaneous image visualisation while analysing and reporting on complex image data sets. However, most analytical software can import image data but does not support viewing and exploring of digital images. Different laboratories are incorporating different downstream software into their workflow to analyse and represent their image data. However, a centralised image analysis pipeline that integrates image acquisition, image processing, image analysis algorithms and data visualisation is critical to help improve scientific reproducibility.

One of the important hurdles in clinical adoption of digital pathology or image analysis is the integration and interoperability of digital pathology. Clinical adoption of digital pathology or image analysis has become increasingly expensive and is labour intensive and time consuming to install and adjust with regard to standards and practices. With this rapid evolution in technology, it is vital that these new hardware and software platforms can be easily implemented and integrated within existing LISs in pathology workflows, and as such that they do not become outdated very quickly. In addition to integration and implementation, there are issues concerning interoperability between the different vendors. Commercial software that comes bundled with a microscope is often convenient to use. Closed hardware and software systems offer less flexibility for third‐party integration. Opening up a hardware and software technology with universal standard for data movement and management may facilitate more rapid biomarker identification, although more closed systems may be required for integration of these workflows in digital pathology. This in turn will play a larger role in enhancing the pathology workflow and most importantly improving patient outcome.

Several new solutions that perform simultaneous high‐throughput fluorescence multiplexing have been developed, each with its own limitations in terms of time, cost, throughput, flexibility and scalability (Table 5). Some of these platforms also provide fully integrated image acquisition and multiplexed data analytics that may overcome current obstacles of integration and interoperability. For example, Nanostring's GeoMx^®^ Digital Spatial Profiling (DSP),^31^, ^32^ Akoya Biosciences CODEX^®^,^33^ Miltenybiotec's MACSima™,^36^, ^37^ and IONpath's MIBIscope™^30^ are some of the emerging platforms that offer end‐to‐end solutions from highly multiplexed staining and image acquisition to a fully integrated high‐plex data analytics. Advances in fully integrated multiplexed methodologies and tissue‐based image analysis solutions that can integrate with clinical digital pathology workflow are critical for delivering better diagnostic and treatment decision to cancer patients.

**Table 5:** Key advantages and disadvantages of emerging high‐plex (> 10) IHC platform

Multiplexed solutions	Key advantages	Key disadvantages
GeoMx^®^ DSP (DNA barcode technology)	40‐plex staining Clinical relevance +700 mRNA *in situ* detection No autofluorescence and spectral overlap Bundled image acquisition and analytical software	Limited ROIs Resolution (10–20 µm) No image construction
CODEX ^®^ (DNA barcode technology)	40‐plex staining No spectral overlap Single‐cell data High resolution Low cost add‐on to existing imaging platforms Bundled image acquisition and analytical software	Low publication record
InSituPlex ^®^ (DNA barcode technology)	>16‐plex Retains tissue integrity Reagent only platform No spectral overlap	Requires manual or existing automated staining platforms Requires existing or third‐party imaging and analysis platform Comparatively low plex
Hyperion (Imaging mass cytometry)	>40 markers simultaneously No autofluorescence and spectral overlap Single‐cell data ~0.5 µm resolution	Number of simultaneous markers is limited to existing heavy metals Low throughput Requires existing or third‐party analysis software
MACSima™ (Imaging cyclic staining technology)	Multiplexing of 100+ antibodies Fully automated all‐in‐one workflow > 1500 validated antibodies Single‐cell data Bundled image acquisition and analytical software	High costs associated with instrument, reagents and antibodies Low publication record
MIBIscope™ (Multiplexed Ion Beam Imaging technology)	>40 markers simultaneously No autofluorescence and spectral overlap Single‐cell data ~0.5 µm resolution Whole slide scanning Bundled image acquisition and analytical software	Number of simultaneous markers is limited to existing heavy metals Low throughput

## Conclusions

There is a huge clinical need to understand immune contexture, particularly in cases where patients are refractory to current immunotherapy, or relapse. Characterising immune cells requires complex phenotyping of various markers that is currently beyond the capabilities of chromogenic IHC which is widely used in the clinic for diagnosis of disease. Advances in fluorescence IHC have made it possible to view multi‐parameter data on the same slide, and advances in analysis have made it possible to provide clinically relevant data from these images. It is also possible to present these data in a format that is currently palatable to clinicians and immunologists, and to use complex phenotypes to identify new potential biomarkers for treatment and tracking of disease progression. With current advances in technology driven by research laboratories and CROs, there is little hampering in the translation of such technologies to meaningful patient outcomes. A new era will see production of better dyes, faster technology, more biomarkers, better segmentation and a faster more reproducible overall workflow.

## Author Contributions

**Reshma Shakya:** Conceptualization; Methodology; Visualization; Writing‐original draft; Writing‐review & editing. **TamHong Nguyen:** Methodology; Visualization; Writing‐review & editing. **Nigel Waterhouse:** Conceptualization; Methodology; Visualization; Writing‐review & editing. **Rajiv Khanna:** Conceptualization; Methodology; Supervision; Visualization; Writing‐review & editing.

## Conflict of Interest

RS is Ambassador for De Novo Software in Australia as a consequence of their collaboration on developing FCS Express Image Plus to open fluorescence files acquired using the Vectra Automated Pathology Platform. RK holds international patents on adoptive immunotherapy and vaccine development, which have been licensed to Atara Biotherapeutics; RK acts as consultants for Atara Biotherapeutics and is on the Scientific Advisory Board of Atara Biotherapeutics.
